# Endoscopic submucosal dissection of challenging rectosigmoid anastomotic lesion made feasible by a multipolar adaptive traction device combined with a line

**DOI:** 10.1055/a-2346-4744

**Published:** 2024-07-08

**Authors:** Elena De Cristofaro, Jérôme Rivory, Louis-Jean Masgnaux, Jean Grimaldi, Fabien Pinard, Timothée Wallenhorst, Mathieu Pioche

**Affiliations:** 1 9318 Gastroenterology Unit, Department of Systems Medicine, University of Rome Tor Vergata, Roma, Italy; 2 Gastroenterology and Endoscopy Unit, Edouard Herriot Hospital, Hospices Civils de Lyon, Lyon, France; 3 55151 Gastroenterology and Endoscopy Unit, Hospital Centre Cornouaille, Quimper, France; 4 Gastroenterology and Endoscopy Unit, Pontchaillou University Hospital, Rennes, France

Endoscopic submucosal dissection (ESD) is widely accepted as a minimally invasive treatment for most superficial colorectal neoplasms, enabling a high rate of en bloc R0 resection 1 . However, ESD is difficult to perform in selected cases, such as in anastomotic lesions following colorectal surgery. In such instances, ESD presents technical challenges due to severe fibrosis, limited space, the presence of staples, deformities, and suture lines from previous surgeries, which increase the risk of complications and unsuccessful outcomes 2 3 .

We have already discussed the benefits of using an adaptative traction device (A-TRACT) in selected difficult lesions 4 5 .

In this case we report the use of an A-TRACT 2+2 associated with a line ( Fig. 1 ) to maximize the effects of traction during dissection of a rectosigmoid anastomotic lesion ( Video 1 ).

**Fig. 1:**
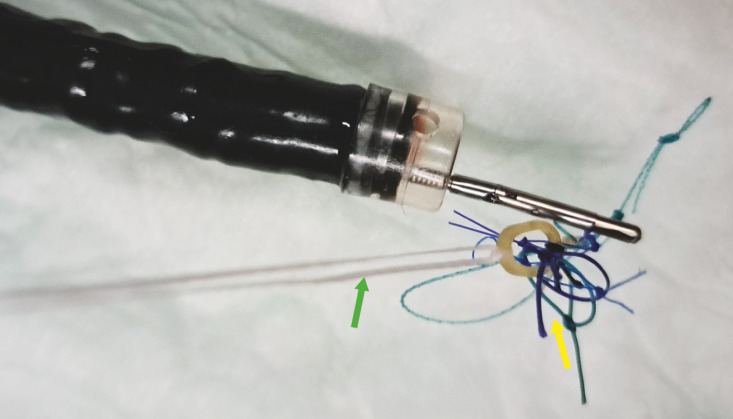
A-TRACT adaptative traction device (yellow arrow) attached to a line (green arrow).

Use of adaptative traction device with a line for a rectosigmoid anastomotic lesion.Video 1

A 76-year-old man was referred for ESD of a 10-mm lesion located at the rectosigmoid anastomosis. After circumferential incision and trimming, the A-TRACT 2+2 was applied by placing the four loops on the lesion’s edges. The line that had previously been attached to the A-TRACT was gently pulled to improve the traction ( Fig. 2 ). Underwater ESD was used during the dissection phase to optimize the visibility of the submucosa and muscularis propria. Any staple encountered during the procedure was either removed or avoided, ensuring the excision line was made below it within the thickened muscularis propria. After cutting half of the lesion, the A-TRACT was tightened, and the line was pulled slightly more to better expose the layers. The procedure was concluded without adverse events. The histopathology revealed an adenoma with high grade dysplasia and free margins.

**Fig. 2:**
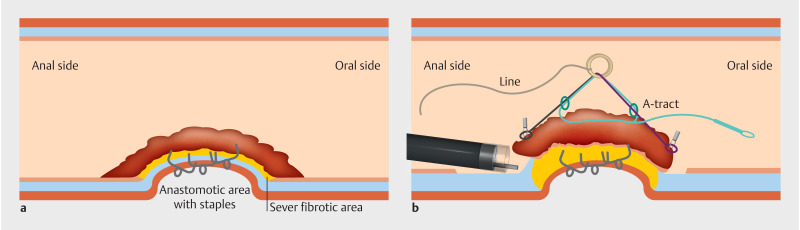
Schematic representation of the procedure using ATRACT 2+2 with a line to maximize the traction during the dissection phase.

We can infer that the use of a line attached to the A-TRACT system offers improved adaptability of traction, potentially making ESD of rectosigmoid anastomotic lesions with limited lumen space safer and more feasible.

Endoscopy_UCTN_Code_TTT_1AO_2AG_3AD

## Notes

Endoscopy E-Videos https://eref.thieme.de/e-videos
*E-Videos* is an open access online section of the journal *Endoscopy* , reporting on interesting cases and new techniques in gastroenterological endoscopy. All papers include a high-quality video and are published with a Creative Commons CC-BY license. Endoscopy E-Videos qualify for HINARI discounts and waivers and eligibility is automatically checked during the submission process. We grant 100% waivers to articles whose corresponding authors are based in Group A countries and 50% waivers to those who are based in Group B countries as classified by Research4Life (see: https://www.research4life.org/access/eligibility/ ). This section has its own submission website at https://mc.manuscriptcentral.com/e-videos .
